# Delayed Disease Progression in Cynomolgus Macaques Infected with Ebola Virus Makona Strain

**DOI:** 10.3201/eid2110.150259

**Published:** 2015-10

**Authors:** Andrea Marzi, Friederike Feldmann, Patrick W. Hanley, Dana P. Scott, Stephan Günther, Heinz Feldmann

**Affiliations:** National Institutes of Health, Hamilton, Montana, USA (A. Marzi, F. Feldmann, P.W. Hanley, D.P. Scott, H. Feldmann);; Bernard-Nocht-Institute for Tropical Medicine, Hamburg, Germany (S. Günther)

**Keywords:** Ebola virus, viruses, West African Ebola virus isolate, Makona strain, Mayinga strain, Ebola hemorrhagic fever, nonhuman primates, cynomolgus macaques, pathogenesis

## Abstract

In late 2013, the largest documented outbreak of Ebola hemorrhagic fever started in Guinea and has since spread to neighboring countries, resulting in almost 27,000 cases and >11,000 deaths in humans. In March 2014, Ebola virus (EBOV) was identified as the causative agent. This study compares the pathogenesis of a new EBOV strain, Makona, which was isolated in Guinea in 2014 with the prototype strain from the 1976 EBOV outbreak in the former Zaire. Both strains cause lethal disease in cynomolgus macaques with similar pathologic changes and hallmark features of Ebola hemorrhagic fever. However, disease progression was delayed in EBOV-Makona–infected animals, suggesting decreased rather than increased virulence of this most recent EBOV strain.

## Abstract 2

Slower progression suggests decreased virulence of this new strain.

Ebola virus (EBOV) was discovered 1976 in central Africa and has since then caused multiple localized outbreaks of Ebola hemorrhagic fever (EHF) in humans and great apes in this area (*1*). In December 2013, an EBOV outbreak started in West Africa, in rural eastern Guinea (*2*). From there the disease spread to the neighboring countries of Liberia, Sierra Leone, and Mali and has been imported into Nigeria, Senegal, Spain, the United Kingdom, and the United States (*3*). Almost 27,000 human cases and >11,000 deaths have been documented, making this the largest EBOV outbreak on record (*3*).

Although no approved treatment or vaccine is available for Ebola, this outbreak emphasizes the urgent need for countermeasures. Recently, several experimental EBOV therapeutics and vaccines have been accelerated for licensure and clinical trials are underway (www.clinicaltrials.gov), underlining the severity of the situation for global public health.

The current EHF outbreak was caused by a new strain of EBOV, designated EBOV-Makona, which was first isolated in Guinea in 2014 (*2*). To determine the virulence of the new EBOV-Makona strain from West Africa in the standard macaque animal model, we infected cynomolgus macaques with EBOV-Makona (*2*) and compared infection with that caused by the prototype EBOV strain Mayinga (EBOV-Mayinga) isolated in 1976 (*1*). These virus isolates are 97% identical in their genomic sequence (*2*). 

## Materials and Methods

### Biosafety and Animal Ethics Statements

All infectious work with EBOV was performed by using standard operating procedures approved by the Rocky Mountain Laboratories (RML) Institutional Biosafety Committee in the maximum containment laboratory at the RML, Division of Intramural Research, National Institute of Allergy and Infectious Diseases, National Institutes of Health (Hamilton, MT, USA). Animal work was performed in strict accordance with the recommendations described in the Guide for the Care and Use of Laboratory Animals of the National Institute of Health, the Office of Animal Welfare and the United States Department of Agriculture. Procedures were conducted in animals anesthetized with ketamine by trained personnel under the supervision of veterinary staff. All efforts were made to ameliorate the welfare and minimize animal suffering in accordance with the Weatherall report on the use of nonhuman primates in research (https://royalsociety.org/policy/publications/2006/weatherall-report/).

Animals were housed in adjoining individual primate cages that enabled social interactions, under controlled conditions of humidity, temperature, and light (12-h light:12-h dark cycles). Food and water were available ad libitum. Animals were monitored at least twice a day and fed commercial monkey chow, treats, and fruit twice a day by trained personnel. Environmental enrichment consisted of commercial toys. Early endpoint criteria, as specified by the RML Animal Care and Use Committee–approved clinical score parameters, were used to determine when animals should be humanely euthanized.

### Challenge Viruses

EBOV-Mayinga (passage 5) (*1*) and EBOV-Makona C07 (passage 1) (*2*) were used. These 2 viruses were propagated on Vero E6 cells, titered on Vero E6 cells, and stored in liquid nitrogen.

### Study Design

Six cynomolgus macaques (*Macaca fascularis*) (4 male and 2 female animals, age range 6–8 years, weight range 3–7 kg) were used in this study (3 animals/group). All animals were challenged intramuscularly at 2 anatomic locations, the left and right caudal thigh, on day 0 with a dose of 1,000 PFU of EBOV-Mayinga or EBOV-Makona. The animals were observed at least twice a day for clinical signs of disease according to an **Institutional Animal Care and Use Committee–**approved and previously published scoring sheet (*4*) and were humanely euthanized when clinical signs indicated terminal disease on the basis of pre-established endpoints. Blood samples were collected on days 0, 3, and 6 after infection and at time of euthanasia. A full necropsy was performed at the end of the study, and organs were harvested for virologic and pathologic analyses.

### Virus Loads

For determination of virus loads in macaque blood and tissue samples, Vero E6 cells were seeded in 48-well plates the day before titration. Tissues were homogenized in 1 mL plain Dulbecco minimal essential medium, and tissue and blood samples were serial diluted 10-fold. Medium was removed from cells and triplicates were inoculated with each dilution. After 1 h, Dulbecco minimal essential medium supplemented with 2% fetal bovine serum, penicillin/streptomycin and l-glutamine was added and incubated at 37°C. Cells were monitored for cytopathic effect, and 50% tissue culture infectious dose was calculated for each sample by using the method of Reed and Muench (*5*).

### Hematologic and Chemical Analyses

Total leukocyte counts; lymphocyte, platelet, reticulocyte, and erythrocyte counts; hemoglobin levels; hematocrit values; mean cell volumes; mean corpuscular volumes, and mean corpuscular hemoglobin concentrations were determined from blood containing EDTA by using the HemaVet 950FS Analyzer (Drew Scientific, Dallas, TX, USA). Serum biochemical analysis was conducted by using the Piccolo Xpress Chemistry Analyzer and Piccolo General Chemistry 13 Panel Discs (Abaxis, Union City, CA, USA).

### Serum Cytokine Levels

Macaque serum samples were inactivated by using γ-irradiation (5 MRad) and removed from the maximum containment laboratory. Serum samples were then diluted 1:2 in serum matrix for analysis by using the Milliplex Non-Human Primate Magnetic Bead Panel (Millipore, Billerica, MA, USA) according to the manufacturer’s instructions. Concentrations of tumor necrosis factor-α, interleukin 6 (IL-6), IL-12/23p40, IL-8, monocyte chemotactic protein 1, IL1Ra, soluble CD40L (sCD40L), IL-15, interferon-γ (IFN-γ), IL-4, and IL-17 were determined for all samples by using the Bio-Plex 200 System (BioRad Laboratories, Hercules, CA, USA).

### Histopathologic and Immunohistochemical Analyses

Analyses were performed with macaque tissues. After fixation/inactivation for 7 days in 10% neutral-buffered formalin, samples were removed from high containment. Subsequently, tissue samples were embedded in paraffin and tissue sections were stained with hematoxylin and eosin. Liver and spleen samples were evaluated in detail, and the following scoring system was applied: 0 = no lesions; 1 = small number of necrotic cells; 2 = moderate necrosis; 3 = significant necrosis; 4 = coalescing necrosis; 5 = diffuse necrosis. To detect EBOV antigen, immunohistochemical analysis was performed by using polyclonal rabbit serum against Ebola virus viral protein 40 as described (*6*).

### Statistical Analyses

A log-rank (Mantel-Cox) test was performed to analyze time to death between the 2 groups. Statistical significance was determined at a level of 0.05. All analyses were conducted by using Prism software (GraphPad Software, San Diego, CA, USA).

## Results

### Disease Progression

**Figure 1:**
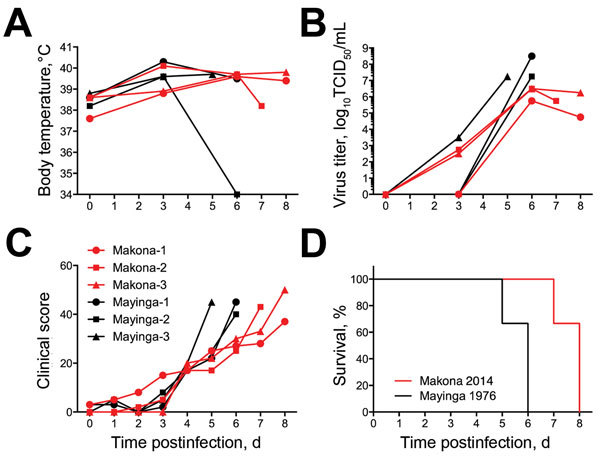
Clinical parameters for 6 cynomolgus macaques infected with Ebola virus strains Makona or Mayinga. Parameters were measured for each animal on day of examination and time of euthanasia. A) Temperature profiles. B) Virus titer (viremia). TCID_50_, 50% tissue culture infectious dose. C) Daily clinical scorel. D) Survival curves showing significant difference in time to death between both groups (p = 0.0295, by Mantel-Cox test).

Six cynomolgus macaques were randomly divided into 2 groups and infected intramuscularly with 1,000 PFU of EBOV-Makona or EBOV-Mayinga. The targeted challenge dose was confirmed by back-titration of the inoculum on Vero cells. On days 3 and 6 postinfection, all macaques were febrile (temperature >38.5°C) (Figure 1, panel A), and animals showed a decrease in food intake and general activity starting at day 4 postinfection. A prominent, macular, cutaneous rash developed in the 3 EBOV-Mayinga−infected animals throughout their bodies starting on day 4 postinfection. In contrast, this characteristic sign of EHF developed in only 2 of the 3 EBOV-Makona−infected animals on day 6, and the rash was more faint and restricted to the arms, legs, chest, and face.

Unlike in current human EHF cases, diarrhea was only observed temporarily in 1 EBOV-Makona−infected and in none of the EBOV-Mayinga−infected animals. Viremia was detectable as early as day 3 postinfection with no clear distinction among the 2 groups, but end titers were in general higher in EBOV-Mayinga−infected animals (Figure 1, panel B). The 3 EBOV-Mayinga−infected animals had to be euthanized on days 5 and 6 postinfection (1 and 2 animals, respectively) because of severity of disease and after reaching the critical clinical score for euthanasia according to approved animal study protocol (Figure 1, panels C, D) (*4*). In contrast, EBOV-Makona infection resulted in slower disease progression; animals were euthanized on days 7 and 8 postinfection (1 and 2 animals, respectively) (Figure 1, panels C, D), a distinction that was significant (p = 0.0295).

### Hematologic and Chemical Analyses

**Figure 2:**
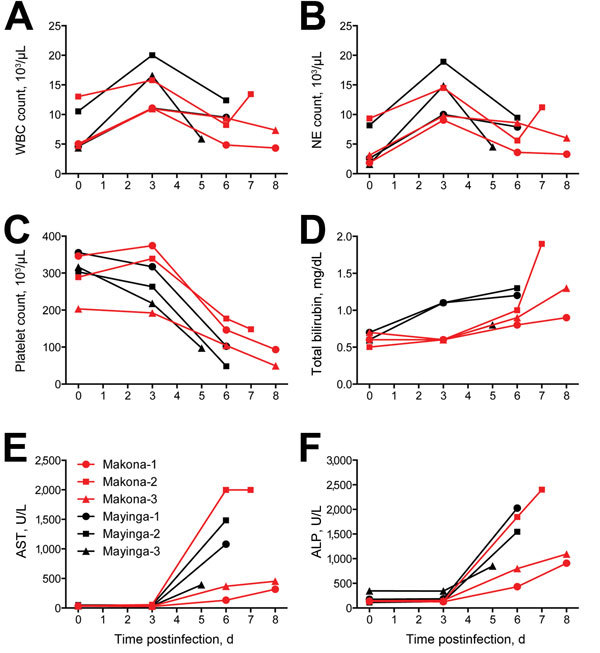
Blood and serum parameters for 6 cynomolgus macaques infected with Ebola virus strains Makona or Mayinga. Parameters were measured for each animal on day of examination and time of euthanasia. A) Leukocyte counts. WBC, white blood cell. B) Neutrophil (NE) counts. C) Platelet counts. D) Bilirubin levels. E) Aspartate aminotransferase (AST) levels. F) Akaline phosphatase (ALP) levels.

An increase in leukocytes (Figure 2, panel A) was noted in all 6 animals on day 3 postinfection with concurrent neutrophilia (Figure 2, panel B) and lymphopenia, a phenomenon that has been reported in EBOV-infected macaques (*7*–*9*). Platelet levels decreased over time in all animals, and thrombocytopenia developed during days 3−6 postinfection (Figure 2, panel C). Although the overall hemogram changes were similar for both groups, EBOV-Makona−infected animals often showed a delay and less severe changes than EBOV-Mayinga−infected animals. Animals in both groups showed mild increases in levels of total bilirubin (Figure 2, panel D) and moderate to severe increases in levels of liver enzymes, such as aspartate aminotransferase (Figure 2, panel E) and alkaline phosphatase (Figure 2, panel F). These findings are consistent with those of acute, diffuse hepatic necrosis, which is commonly found with EBOV infection in macaques (*7*–*9*). Again, increases in liver enzyme levels were delayed in 2 of the 3 EBOV-Makona−infected animals and did not always reach the levels for EBOV-Mayinga−infected animals.

### Systemic Host Responses

**Figure 3:**
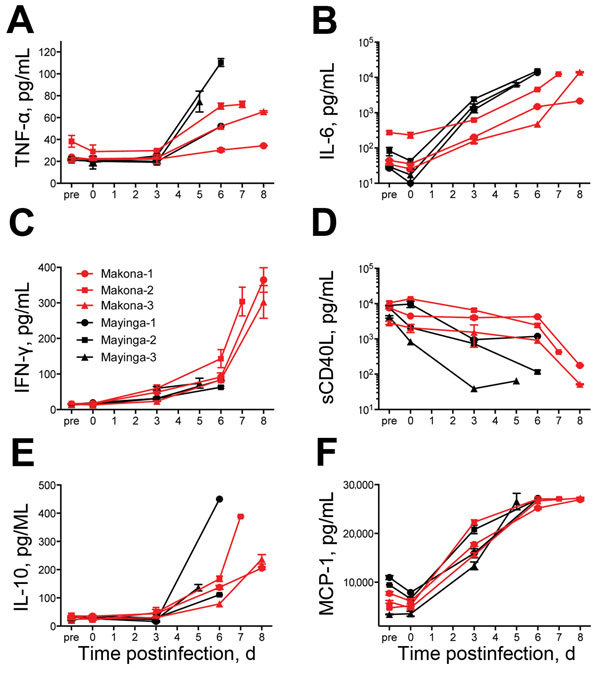
Serum cytokine and chemokine levels for 6 cynomolgus macaques infected with Ebola virus strains Makona or Mayinga. A) Tumor necrosis factor-α (TNF-α); B) Interleukin-6 (IL-6); C) Interferon-γ (IFN-γ); D) Soluble CD40 ligand (sCD40L); E) IL-10; and F) Monocyte chemotactic protein 1 (MCP-1). Kinetics were analyzed in serum samples of each animal collected on days of examination and time of euthanasia.

Differences in systemic host immune responses were monitored in serum of infected animals. Levels of some proinflammatory cytokines increased in later stages of the disease in all animals (Figure 3). Although levels of tumor necrosis factor-α were similar (slightly higher for EBOV-Mayinga−infected animals) for both groups over time (Figure 3, panel A), serum levels of IL-6 increased earlier in the EBOV-Mayinga−infected animals and reached similar levels at time of euthanasia (Figure 3, panel B). The most striking difference was observed for IFN-γ; a >300% increase was observed in EBOV-Makona−infected animals at the time of euthanasia (Figure 3, panel C). Thrombocytopenia is a hallmark of EHF in human and nonhuman primates. Therefore, we monitored levels of sCD40L, a marker for platelet activation. Levels of sCD40L decreased in both groups during the infection (Figure 3, panel D), a fact that correlated well with decreasing platelet levels (Figure 3, panel C). Finally, antiinflammatory marker IL-10 and chemokine monocyte chemotactic protein 1 were found to be upregulated during the course of infection in all the animals, and there were no differences between the 2 groups (Figure 3, panels E, F). Overall, targeted systemic host responses were upregulated for all the animals, and except for IFN-γ, there were minor differences between the 2 groups and among individual animals.

### Pathologic Changes

**Figure 4:**
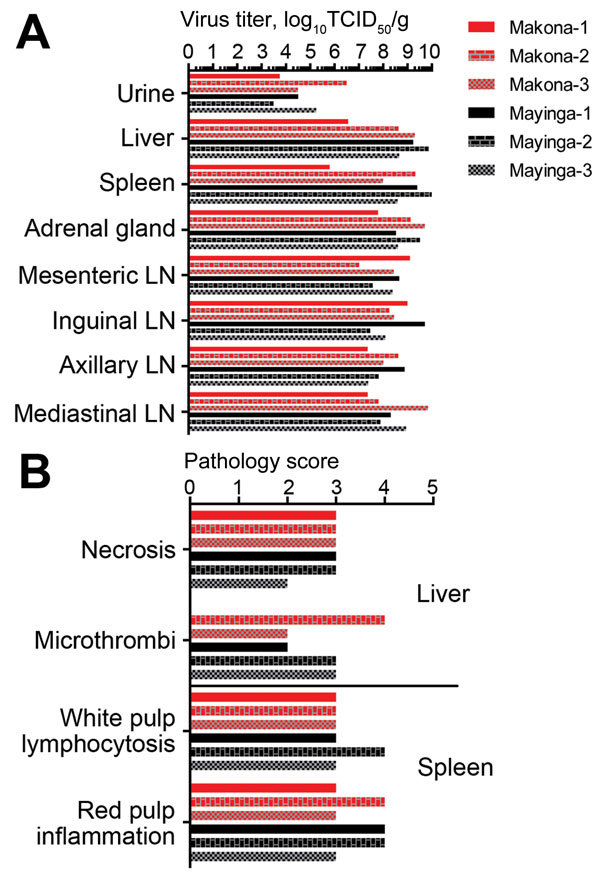
Virologic and pathologic results for 6 cynomolgus macaques infected with Ebola virus strains Makona or Mayinga. A) Viral infectivity titers were determined in key tissue samples collected at the time of euthanasia. TCID_50_, 50% tissue culture infectious dose; LN, lymph node. B) Pathology scores for liver and spleen. Scores were generated by using the scoring system described in the Materials and Methods.

**Figure 5:**
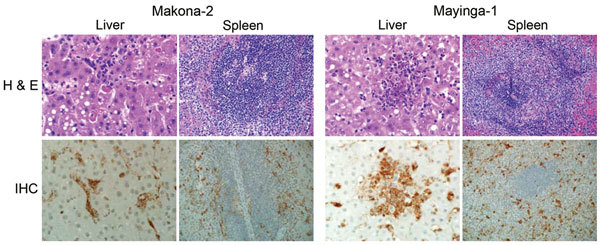
Pathologic results for 6 cynomolgus macaques infected with Ebola virus strains Makona or Mayinga. Liver and spleen sections were stained with hematoxylin and eosin (H & E; top panels) and analyzed for necrosis, microthrombi, lymphocytosis, and inflammation. Sections were also stained with a polyclonal rabbit serum against Ebola virus viral protein 40 for detection of viral antigen (immunohistochemical [IHC] analysis; bottom panels). Sections from a representative animal in each group are shown. Original magnification levels: liver, ×40; spleen, ×20.

Animals in both groups showed typical gross pathologic changes for EBOV infections in macaques, and no differences in virus titers were found in tissues collected at the time of euthanasia (Figure 4, panel A). Pathology scores for liver and spleen, key target organs for EBOV infection, were similar (Figure 4, panel B). There were no apparent histopathologic differences between lesions induced by EBOV-Makona or EBOV-Mayinga infections, and both strains caused multifocal to coalescing hepatic necrosis with acute inflammation (Figure 5, upper panels). In addition, there were abundant fibrin microthrombi within the hepatic sinusoids. Likewise, splenic lesions produced by either virus strain were indistinguishable and consisted of necrosis (lymphocytolysis) of the white pulp, abundant fibrin within the red pulp, and multifocal acute inflammation (Figure 5, upper panel). Immunohistochemical analysis showed copious viral antigen in hepatocytes, endothelial cells, and mononuclear cells, which was collocated with hepatic and splenic lesions (Figure 5, lower panels). Overall, lethal EHF developed in all animals, and they showed similar anatomic pathologic features.

## Discussion

EHF with similar hallmarks of the disease developed in all infected cynomolgus macaques, and all animals had to be euthanized according to protocol. However, despite similar onset of symptoms (fever) for all animals, disease progression in the EBOV-Makona–infected animals was delayed, which suggested attenuation in virulence for this strain in macaques. Nevertheless, the macaque model is well suited to determine the efficacy of existing vaccines (historically assessed in cynomolgus macaques) and treatment strategies (historically tested in rhesus macaques) against the currently circulating EBOV-Makona strain in West Africa.

In this study, we sought to establish a macaque disease model for the EBOV-Makona strain currently circulating in West Africa to advance our response capabilities by testing urgently needed countermeasures against this newly emerged virus and by that fulfilling a critical capacity gap. We compared disease progression and pathogenesis of 2 EBOV strains, the prototype EBOV-Mayinga and the current EBOV-Makona strain (*2*), in cynomolgus macaques. In general, infections in both groups were similar with regard to disease onset, hematology, blood chemistry, systemic host responses, and end-stage viral organ load and pathologic changes. However, EBOV-Makona–infected animals lagged behind in many parameters; the most notable difference was in progression to end-stage disease. Although clinical scores remained similar in the early stage of disease (until day 4 postinfection), EBOV-Makona−infected macaques reached euthanasia criteria on average 2 days later than EBOV-Mayinga−infected animals (7.67 days vs. 5.67 days postinfection, respectively). This finding was associated with less pronounced and more restricted macular cutaneous rash, slightly lower viremia at end-stage disease, less pronounced increases in liver enzyme levels, and slightly delayed systemic proinflammatory and chemokine responses for EBOV-Makona–infected macaques than for EBOV-Mayinga−infected macaques. Overall, virulence of EBOV-Makona seemed to be decreased in this animal model, although severe/lethal EHF eventually developed in all EBOV-infected macaques. The fact that all infections were lethal explains why end-stage pathologic changes were similar in animals infected with both virus strains and why there were virtually no differences in gross pathologic and histopathologic changes in major target organs.

A major distinction is related to IFN-γ levels during end-stage disease, with dramatically higher systemic levels for EBOV-Makona–infected macaques. This finding has been reported for an EBOV-Mayinga−infected rhesus macaque (*7*) and was described for lethal EHF cases in humans (*10*). EBOV-Mayinga−infected macaques, which showed faster disease progression, did not show these strikingly increased IFN-γ levels. One can only speculate that >7 days (expected time for initiation of adaptive immune responses) after EBOV infection seem to be necessary for adequate stimulation of T cells, the main source of IFN-γ (*11*).

The case-fatality rate in humans in the current outbreak in West Africa (≈50%) does not seem nearly as high as that reported for the initial 1976 outbreak in the former Zaire (now Democratic Republic of the Congo) (≈90%), which suggests lower virulence of this new strain from West Africa (*1*,*3*,*12*,*13*). It is difficult to say if the seemingly lower virulence in humans is reflected in our macaque model because this model remains lethal for all studied EBOV strains, including the current strain in West Africa (EBOV-Makona). However, it seems fair to conclude that virulence of the strain from West Africa in macaques is not increased compared with other EBOV strains. Nevertheless, the macaque model that we developed fills a major capacity gap and can be used for development of urgently needed countermeasures against the new EBOV-Makona strain from West Africa.

## References

[1] Feldmann H, Geisbert TW. Ebola haemorrhagic fever. Lancet. 2011;377:849–62 and. 10.1016/S0140-6736(10)60667-8 21084112 PMC3406178

[2] Baize S, Pannetier D, Oestereich L, Rieger T, Koivogui L, Magassouba N, Emergence of Zaire Ebola virus disease in Guinea. N Engl J Med. 2014;371:1418–25 . 10.1056/NEJMoa1404505 24738640

[3] World Health Organization. Ebola situation report, May 13, 2015. Geneva: The Organization; 2015.

[4] Brining DL, Mattoon JS, Kercher L, LaCasse RA, Safronetz D, Feldmann H, Thoracic radiography as a refinement methodology for the study of H1N1 influenza in cynomologus macaques (*Macaca fascicularis*). Comp Med. 2010;60:389–95 . 21262125 PMC2958208

[5] Reed LJ, Muench H. A simple method of estimating fifty percent endpoints. Am J Hyg. 1938;27:493–7.

[6] Groseth A, Marzi A, Hoenen T, Herwig A, Gardner D, Becker S, The Ebola virus glycoprotein contributes to but is not sufficient for virulence in vivo. PLoS Pathog. 2012;8:e1002847. 10.1371/journal.ppat.1002847 22876185 PMC3410889

[7] Ebihara H, Rockx B, Marzi A, Feldmann F, Haddock E, Brining D, Host response dynamics following lethal infection of rhesus macaques with Zaire ebolavirus. J Infect Dis. 2011;204(Suppl 3):S991–9. 10.1093/infdis/jir336 21987781 PMC3189992

[8] Geisbert TW, Hensley LE, Larsen T, Young HA, Reed DS, Geisbert JB, Pathogenesis of Ebola hemorrhagic fever in cynomolgus macaques: evidence that dendritic cells are early and sustained targets of infection. Am J Pathol. 2003;163:2347–70. 10.1016/S0002-9440(10)63591-2 14633608 PMC1892369

[9] Martins K, Cooper C, Warren T, Wells J, Bell T, Raymond J, Characterization of clinical and immunological parameters during Ebola virus infection of rhesus macaques. Viral Immunol. 2015;28:32–41. 10.1089/vim.2014.0085 25514385

[10] Villinger F, Rollin PE, Brar SS, Chikkala NF, Winter J, Sundstrom JB, Markedly elevated levels of interferon (IFN)-gamma, IFN-alpha, interleukin (IL)-2, IL-10, and tumor necrosis factor-alpha associated with fatal Ebola virus infection. J Infect Dis. 1999;179(Suppl 1):S188–91. 10.1086/514283 9988183

[11] Kurtulus S, Tripathi P, Hildeman DA. Protecting and rescuing the effectors: roles of differentiation and survival in the control of memory T cell development. Front Immunol. 2012;3:404. 10.3389/fimmu.2012.00404 PMC3552183 23346085

[12] Bah EI, Lamah MC, Fletcher T, Jacob ST, Brett-Major DM, Sall AA, Clinical presentation of patients with Ebola virus disease in Conakry, Guinea. N Engl J Med. 2015;372:40–7. 10.1056/NEJMoa1411249 25372658

[13] Schieffelin JS, Shaffer JG, Goba A, Gbakie M, Gire SK, Colubri A, Clinical illness and outcomes in patients with Ebola in Sierra Leone. N Engl J Med. 2014;371:2092–100. 10.1056/NEJMoa1411680 25353969 PMC4318555

